# Nucleolar Organizer Regions in Oral Squamous Cell Carcinoma

**DOI:** 10.5681/joddd.2012.004

**Published:** 2012-03-13

**Authors:** Monir Moradzadeh Khiavi, Sepideh Vosoughhosseini, Monire Halimi, Seyyed Mostafa Mahmoudi, Asghar Yarahmadi

**Affiliations:** ^1^Assistant Professor, Department of Oral Pathology, Faculty of Dentistry, Tabriz University of Medical Sciences, Tabriz, Iran; ^2^Associate Professor, Department of Oral Pathology, Faculty of Dentistry, Tabriz University of Medical Sciences, Tabriz, Iran; ^3^Assistant Professor, Department of Pathology, Imam Reza Hospital, Tabriz University of Medical Sciences, Tabriz, Iran; ^4^Postgraduate Student, Department of Oral Pathology, Faculty of Dentistry, Tabriz University of Medical Sciences, Tabriz, Iran; ^5^Dentist, Private Practice, Tabriz, Iran

**Keywords:** AgNORs, epithelium, squamous cell carcinoma

## Abstract

**Background and aims:**

Several diagnostic methods are being employed to detect benign and malignant lesions, one of which is silver nitrate staining for organizer regions. The number of nucleolar organizing regions (NORs) can be used to show the degree of cell activity or metabolism in pathologic lesions. This study was designed to evaluate NORs as determi-nants of precancerous and squamous cell carcinoma.

**Materials and methods:**

A silver colloid technique was applied on paraffin sections of 40 cases of oral squamous cell carcinoma and 25 cases of precancerous lesions; 15 specimens of normal epithelium were selected for the control group. After staining with silver nitrate, argyrophilic nucleolar organizer regions (AgNORs) were counted in 100 epithelial cells in three groups with the use of an oil immersion and ×1000 objective lens. One-way ANOVA and a post hoc Tukey test were used for statistical analysis.

**Results:**

The mean numbers and standard deviations of AgNORs were 1.58 ± 0.76 in normal epithelium, 2.1 ± 1.05 in pre-cancerous lesions and 2.43 ±1.33 in squamous cell carcinoma (SCC). There were statistically significant differences in Ag-NORs numbers between the groups (P<0.001) and significant differences in precancerous lesions between dysplastic and non-dysplastic epithelia (P<0.001). The mean AgNORs count per nucleus increased from healthy epithelium to precancer-ous lesion to SCC.

**Conclusion:**

This study suggests that the silver staining technique for the detection of NORs (AgNOR) can be used to distinguish precancerous lesions and benign and malignant lesions.

## Introduction

The incidence of oral cancer shows considerable geographical, cultural and ethnic variations. This variation ranges from a low incidence of 1–2% of all malignant tumors in much of Japan to over 40% in Sri Lanka, approaching 50% in India.^1^In one study in Kerman Province in Iran, oral and pharyngeal cancer was the seventh most common cancer of all malignancies. The majority of oral and pharyngeal cancers (71.3%) were squamous cell carcinomas (SCC). A total of 91.6% of squamous cell carcinomas of these regions occurred in the oral cavity.^2^ In general, oral cancer accounts for less than 3% of all cancers. However, squamous cell carcinoma is the most common epithelial malignancy in the oral cavity and it constitutes approximately 94% of all oral malignancies.^3^ Currently, most human cancers are diagnosed based on biopsy and histological examinations with hematoxylin and eosin staining. However, in some instances, hematoxylin and eosin staining is not sufficient to determine the different histopathologic grades of tumors and identify dysplastic changes in precancerous lesions. Thus, AgNOR staining which is cheaper than other staining techniques can be helpful in providing more information about cellular status.^4^ Nucleolar organizer regions (NORs) are loops of DNA transcribed into ribosomal RNA, finally resulting in ribosome and protein formation.^5^They exist on the short arms of the fifth acrocentric chromosomes.^5 - 7^ NORs can be visualized as black dots under a light microscope with high magnification.^7 , 8^In this study, the diagnostic value of silver nitrate staining of NORs in oral SCC, precancerous lesions and normal oral mucosa was evaluated; in addition, the relation between AgNOR numbers in three groups mentioned above was investigated.

## Materials and Methods

The protocol of the present study was approved by the Research and Ethics Committees of Tabriz University of Medical Sciences. In this descriptive double-blind study, 40 paraffin blocks of SCC and 25 precancerous lesions including leukoplakia, erythroplakia and actinic cheilosis based on Hematoxylin and Eosin staining were selected from the Pathology Archives of Imam Reza Hospital. Fifteen specimens of normal epithelium were selected for the control group. The samples were cut into 4-µm-thick slices and AgNOR staining was carried out by Modified Poloton staining method.^9^ First, the samples were dewaxed in xylene and then rehydrated through graded ethanols to distilled water. The silver nitrate solution was prepared by mixing 2 gr of gelatin in 100 mL of 1% formic acid with two parts of 50% silver nitrate solution in distilled water. The sections were incubated in this solution for 60 minutes at room temperature in the dark and then washed in deionized water. This was followed by sequential dehydration in graded alcohol solutions, cleared in xylene and mounted in Canada balsam. AgNORs were seen as distinct intranuclear black dots and were randomly counted manually in 100 nuclei under ×1000 magnification with oil immersion in the three groups. Finally, the mean value and standard deviation of each case were determined. One-way ANOVA was used to compare the three groups. The means of AgNORs were compared in each group with the two other groups by a post hoc Tukey test.

## Results

As shown in Table 1, the means of AgNORs in SCC, precancerous lesions and normal epithelium were 2.43±1.33, 2.1±1.05 and 1.58±0.76, respectively. According to one-way ANOVA, a significant difference was seen in the number of AgNOR dots between the groups (P<0.001). Furthermore, a significant difference was seen in comparison of each group with two other groups by post hoc test (P<0.001) (Table 1). The means of AgNORs in non-dysplastic and dysplastic epithelia were 1.73±0.82 and 2.28±1.1, respectively. There was also a significant difference between non-dysplastic and dysplastic epithelia (P<0.001) according to t-test.

**Table 1:** Means and standard deviations of AgNOR counts in the nucleoli of different types of lesions and normal epithelium

Tissue Type	Number of samples	Mean	Std. Deviation
Normal epithelium ^ a ^	15	1.5813	0.7623
Precancerous lesions (total) ^ b ^	25	2.1072	1.0515
Squamous cell carcinoma ^ c ^	40	2.4338	1.3387

According to post hoc Tukey test differences of a with b and c, b with a and c and also c with a and b were significant (P<0.001).

**Figure 1:**
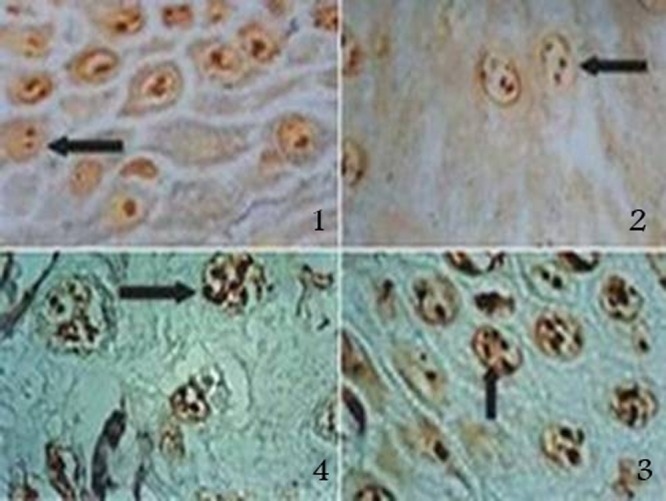
The AgNOR dots with ×1000 magnification in: (1) normal epithelium with two lighter dots than the others (arrow); (2) dysplastic epithelium with three dots (arrow); (3) well-differentiated SCC with darker dots; and (4) moderately differentiated SCC with larger dark dots than the others (arrow).

On the other hand, as an accessory finding AgNORs dots were lighter in normal and dysplastic epithelia compared to squamous cell carcinoma. In squamous cell carcinoma the dots were larger compared to precancerous lesions and also larger in precancerous lesions compared to normal epithelium ( Figure 1).

## Discussion

Although conventional histological staining with hematoxylin and eosin may be useful in determination of dysplastic changes in precancerous lesions and grading of squamous cell carcinoma, sometimes it is difficult to differentiate these lesions with this staining technique. In such cases AgNORs staining seems to be useful. It has been established that quantification of interphase AgNORs can actually represent a valuable tool for cell kinetics evaluation.^10^ Interphase AgNOR accumulation in cells entering the mitotic cycle is associated with an increased request of ribosomal biogenesis. Protein synthesis is faster in rapidly dividing cells as compared to slowly proliferating ones. Therefore, an increase should occur in the nucleolar structures (AgNORs) where rRNA synthesis takes place. For these reasons, the AgNOR parameter has been suggested as a reliable marker for the evaluation of the rate of cell proliferation in routinely processed histologic samples.^10 , 11^A lot of studies have been carried out to determine AgNOR numbers in normal, benign, borderline, and malignant conditions.^5 - 8 , 10 , 13 - 18^ Therefore, the AgNOR counts could be used as a useful marker for investigating nuclear and cellular proliferative activities.^5^ In the present study, a significant statistical difference was seen between normal epithelium, precancerous lesions and squamous cell carcinoma, which shows AgNOR counts can be useful in differentiation of benign and malignant lesions. Crocker et al^12^ first used this technique to assess grades of non-Hodgkin’s lymphoma, and reported that the mean number of AgNORs per nucleus of high-grade lymphomas was much greater than that in low-grade non-Hodgkin's lymphomas. Chattopadhyay et al^13^ studied AgNOR counts in the epithelia of oral buccal mucosa, oral leukoplakia, and oral squamous cell carcinoma (SCC) and a significant statistical difference was seen between normal epithelium and leukoplakia, normal epithelium and squamous cell carcinoma and also between leukoplakia and squamous cell carcinoma. AgNOR counts in squamous cell carcinoma were more than others.The same results have been obtained between aggressive and non-aggressive lesions^14^and also other benign and malignant lesions.^15^ Silva et al^16^ showed a direct association between the number of NORs in OSCC and histologic tumor grade. Ohno et al^15^ reported significant differences between benign and malignant bone tumors and also between benign tumors and normal tissues.^15^ However, in spite of positive correlation between AgNOR counts and cellular proliferation in most studies,^13 - 17^one study showed that AgNOR counts are non-contributory to the diagnosis of dysplastic lesions.^18^ As mentioned above, the nucleolar organizer regions were lighter in normal epithelium and dysplastic epithelium compared to squamous cell carcinoma. The AgNORs were larger in squamous cell carcinoma than precancerous lesions and also larger in precancerous lesions than normal epithelium. These findings are supported by a study carried out by Cabrini.^17^ The next works will focus on determination of correlation between AgNORs and prognosis of squamous cell carcinoma.

## Conclusion

The mean AgNOR count can be a useful tool in definitive diagnosis of epithelial dysplasia and squamous cell carcinoma.
